# Whole Sets of Teeth

**Published:** 1851-01

**Authors:** W. E. Ide

**Affiliations:** Columbus, O.


### WHOLE SETS OF TEETH.

NUMBER IV.

BY W. E. IDE, M.D., COLUMBUS, O.

In No. 3 was described my method of taking *impressions* and getting up the *metallic dies.* On that point I would only say further, that the inexperienced may sometimes find difficulty in so manageing the pouring of the type mettle for the counter die, as to prevent a union with the other cast. The directions before given, with a little practice, will usually be sufficient; but when they are not, all danger of that kind of accident will be avoided by the use of the *fusible metal* for the counter-cast. In case the jaw is more prominent on the alveolar border than above on the labial surface as is not unfrequently the case, the difficulty of *drawing* may be avoided by making the counter-cast in two or more pieces. This is readily accomplished, by imbeding the *perfecting* cast in sand, in such a manner as to receive the metal for the counter-cast on but part of its surface at a time. Two pieces will in nearly all cases be found sufficient, and they can be kept together by being screwed into a large vice. If a *perfect* job is desired, I would in no case or any manner, use the pliars to adapt the plate. When a perfect adaptation of the plates is secured, we would next proceed to make an *articulating model.* To aid in procuring this, we always keep on hand a pair of rather strong coiled wire springs. A rim of wax prepared with mastic is moulded up on each plate, to represent as nearly as possible in altitude the length of the teeth to be inserted—or perhaps leaving it a little higher, especially at the back part. The coiled springs having eyes in each, are attached about midway from the medium line and the rear extremity of the plate to the wax, by blunt pins. The wax being cold and wet by immersing in water is introduced with the plate and springs attached into the mouth. The patient is then desired to move the lower jaw in every direction, till the plates are pressed by the springs into their proper resting place, when the jaws should be carefully brought into their proper relative position. The rim of wax being purposely a little too much elevated on the back part, (when the position of the plate cannot otherwise be seen—when the jaws are closed,) is pared off till each respective rim comes in contact in front, and an equal pressure is observed at all points. The jaws and lips are then closed till the face is observed to have its proper length. Then the sides of the two rims are cut down to correspond with each other, and a perpendicular line is drawn with a pointed instrument through each in front and on each side. The jaws are again moved in every direction, and again brought in opposition. If on doing this several times, the perpendicular lines in the wax are still found to correspond, we are certain of having the exact relative position of the jaws. This is a great desideratum, and a failure in accomplishing it has spoiled many, otherwise well constructed sets of teeth.

On removing the plates, wax, &c., from the mouth, the springs are removed, and the two pieces being restored to their proper relative position, are placed on a table with the rear side against a bit of board about an inch thick and of convenient size. This board being oiled, plaster of Paris mixed as usual with water, is poured upon the upper plate, and extending three or f ur inches back upon the board. After waiting sufficiently long for the hardening process, the board is removed and furrows cut in the corresponding portion of plaster.

This then, being oiled, is inverted and more plaster poured upon it, including, of course, the lower plate. After the plaster is set, the two pieces are separated, the wax removed from the plates, and the whole neatly trimmed. The medium line having been previously marked on the plates, the teeth are selected, ground and fitted in such a manner that the points of the bicuspides and cuspidati shall not come in opposition, but interlock each other—the upper cuspidatus striking between the lower cuspidatus and bicuspid. If springs are used, (which are rarely necessary,) they should be attached to a pin passing through the rear bicuspid of the upper plate, and to one passing through a gold tube inserted between the rear bicuspid and first molar of the lower plate.

The end of this tube pointing toward the center is soldered to the linings of the teeth, between which it is passed. In getting the relative bearing of the upper and lower bicuspid and molar teeth, the point of greatest pressure should be directly upon or *within* a line corresponding to the greatest prominence of the alveolar border, if possible. To accomplish this, it often becomes necessary to *pitch* the lower teeth somewhat towards the center, and also, to have their inner edges on the grinding surface somewhat more elevated than the outer. If the line of pressure falls *without* the alveolar ridge in dental occlusion, it requires but slight mechanical knowledge to enable us to see that the usefulness of pieces in mastication must be greatly impaired. The teeth being arranged on the principles above described, and made secure in their places by means of the bees-wax and mastic preparation, are removed from the model with the plates, and if convenient, they can be tried in the mouth. If every previous step has been taken with sufficient care and skill, this is not a matter of importance; as the proper bearing has been secured by the model and the relative length of the front teeth, their color with reference to complexion, age, &c., it is supposed, has been duly noted at the time of preparing the model. Each tooth, spring-holder, &c., being perfectly in place, a thin cast iron ring, of a diameter one-third or one-half greater than the plate, and an inch and a quarter deep, is half filled with a batter of one-third clean coarse river sand, and two thirds fine strong plaster. Into this, the concave surface of the plate is introduced, and between it and the ring is placed a circle of coiled and stiff iron wire, when more of the plaster and sand mixture is poured on until the whole is imbedded some little distance above the teeth. The coiled or louped circlet of wire is a mattter of importance; as when it is used, the teeth can in no way be displaced by the *cracking* of the plaster. The plaster and sand must also be used immediately after mixing with water, or the hardening process may be impaired. When this process has been accomplished, the wax may be removed from the inner surface of the teeth, by warming over a spirit-lamp to soften any remaining particles. The platina pins and plate must be scoured by a common tooth-brush and pumice-stone, and then brushed over with a solution of borax.

In putting on the back-linings, the teeth should *in no case* be removed from their bed in the plaster. To prevent any possibility of their even getting loose, the plaster should come over their ends sufficiently to make them perfectly firm. We next cut strips of gold, in width corresponding with the teeth, and considerably thicker than the palate plate. One end of the strip is cut with a file to fit the main plate at the base of the tooth to which it is to be attached, and being pressed down against its resting place, is moved gently against the lower platina pin. A slight mark is made by it, and at that point it is pierced by a punch. The position for the upper pin is marked in a similar way and pierced, and the back-lining cut of proper length, smoothed with a file, and pressed to its place. The pins are never to be riveted, but *split* with a small gouge-shaped instrument, and cut off, taking care that they nearly, but not quite, fill the holes in the lining plate. When all the teeth are treated in this manner, taking care that all parts to be soldered are in contact, they are again brushed over with the borax solution.

Pieces of solder being placed on each pin and all other points that are to be united, the whole is to be covered by a piece of iron plate, and put into a charcoal furnace. The heat should be very gradual till it reaches a cherry-red, when the piece is to be removed and a well directed stream from a common spirit-lamp of large size, and a mouth blow-pipe, will in a very short time complete the soldering process. The piece may then be returned to the furnace, when the fire has become somewhat deadened, or buried in hot ashes and cooled very gradually. The imbedding in a large quantity of plaster and sand, the binding together by the coiled wire, the gradual and even application and diminution of heat, I consider worth all the *molasses specifics* ever discovered, for preventing the warping or springing of plates. Using these precautions, a plate cannot spring *much*. If it does so at all, it must be restored by placing the piece on the cast, and making a ribbon of wax about an inch wide, and attaching it all around to the plate and linings of the teeth, and transversely across the rear of the plate. After flangsing the wax when it projects above the teeth, pour in plaster till the wax cup is full. Withdrawing this, next mould it in sand, and cast in lead. Removing the plate of teeth from the die, swedge the lead on the corresponding part of the die, till there is a perfect adaptation. Then replace the plate on the die, and drive the lead gently upon it. In this way every part of the plate is pressed upon, except that occupied by the teeth; and although the lead comes very near, yet it does not touch or endanger them.

It will be found that by this careful manner of soldering and adjusting the back-linings, the finishing process becomes a very simple one.

First, use a file till an even surface is obtained. Then with a bit of soft wood moistened in water, and dipped in pulverized pumice-stone, *scour* the whole surface; after which, rub with Scotch stone, and proceed to use the brush and rotten-stone mixed with sweet oil. The rotten-stone is to be finely pulverized and mixed with the oil to the consistence of paste, and with a small spatula put upon the plate, and the brushing continued in a lathe, till a perfectly smooth surface is obtained. After thoroughly washing with soap and water, and wiping dry, take red oxide of iron or *crocus*, and mix with alcohol to a thin paste, and apply as the rotten-stone and oil was done, but of course using another brush. A moment's time only is required in using this, and a beautiful polished surface is presented. Again wash in soap suds and the piece is completed. Infinitely less time is required, a more beautiful polish is produced, and less danger of springing the plate is incurred, than when a burnisher is used.

